# Scalable Synthesis of Pt Nanoflowers on Solution‐Processed MoS_2_ Thin Film for Efficient Hydrogen Evolution Reaction

**DOI:** 10.1002/smsc.202200043

**Published:** 2022-08-02

**Authors:** Yun Seong Cho, Dongjoon Rhee, Jeongha Eom, Jihyun Kim, Myeongjin Jung, Youngdoo Son, Young-Kyu Han, Ki Kang Kim, Joohoon Kang

**Affiliations:** ^1^ School of Advanced Materials Science and Engineering Sungkyunkwan University (SKKU) Suwon 16419 Republic of Korea; ^2^ Department of Industrial and Systems Engineering Dongguk University-Seoul Seoul 04620 Republic of Korea; ^3^ Department of Energy and Materials Engineering Dongguk University-Seoul Seoul 04620 Republic of Korea; ^4^ Department of Energy Science Sungkyunkwan University (SKKU) Suwon 16419 Republic of Korea; ^5^ Center for Integrated Nanostructure Physics (CINAP) Institute for Basic Science (IBS) Sungkyunkwan University (SKKU) Suwon 16419 Republic of Korea; ^6^ KIST-SKKU Carbon-Neutral Research Center Sungkyunkwan University (SKKU) Suwon 16419 Republic of Korea

**Keywords:** electrochemical water splitting, electrodeposition, platinum nanocatalysts, solution processing, 2D materials

## Abstract

Nanostructuring of Pt nanocatalysts increases the surface‐to‐volume ratio, thus enabling efficient usage of Pt for hydrogen evolution reaction (HER). Direct electrochemical reduction of Pt on the electrode can produce nanostructured Pt catalysts, which has been time‐consuming for the conventional colloidal synthesis. However, carbon‐based growth templates commonly used to create Pt nanoparticles offer limited control over morphologies and HER performance. Herein, a facile electrochemical synthesis of Pt nanoflowers (NFs) with well‐defined petals is presented. Semiconducting MoS_2_ nanosheets are solution processed into a film on a carbon paper (CP) to synthesize Pt NFs upon reduction of Pt precursor. The Pt NFs show higher HER activities than spherical or spiky Pt nanoparticles because of their larger active surface area and enable faster release of hydrogen bubbles during HER. By generating sulfur vacancies and MoO_*x*_ on the MoS_2_ template using a reactive ion etching, the areal density and spatial uniformity of Pt NFs can be greatly enhanced and a mass activity can be achieved more than 10 times as high as that of the conventional Pt/C electrode. Multiple electrodes with nearly similar electrochemical properties can be repeatedly produced by using a single precursor solution, which highlights the cost‐efficiency and scalability of our synthesis strategy.

## Graphical abstract

High‐throughput, scalable method to synthesize flower‐like Pt nanoparticles (Pt nanoflowers [NFs]) is developed using semiconducting MoS_2_ nanosheet film as a template for electrochemical reduction of Pt precursors. The resulting Pt NFs exhibit enhanced hydrogen evolution reaction (HER) activities than spherical or spiky Pt nanoparticles as well as operational stability benefiting from the nanoscale morphology.

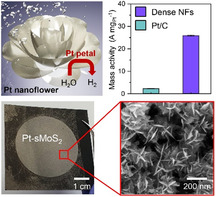

## 1 Introduction

Intensive use of fossil fuels has resulted in carbon dioxide (CO_2_) emissions exceeding the uptake capacity of the natural carbon sinks and substantially contributed to global warming.^[^ ^1^ ^]^ To mitigate the global increase in temperature, achieving carbon neutrality—a state where the amount of CO_2_ emission is equal to the amount absorbed—by decreasing fossil fuel consumption is becoming increasingly important.^[^ ^1^, ^2^ ^]^ Hydrogen has drawn significant interest as a clean energy source due to its zero‐carbon content and high energy density;^[^ ^3^ ^]^ however, most of the hydrogen production is via steam reforming, which consumes natural gas and generates CO_2_.^[^ ^4^ ^]^ To take the full advantage of hydrogen for reducing atmospheric CO_2_ levels, electrochemical water splitting has been extensively investigated because the reaction can be powered by carbon‐free, renewable energies such as sunlight and wind.[^4^, ^5^] To drive the electrolysis of water with a low power supply, electrocatalysts exhibiting a low overpotential for hydrogen evolution reaction (HER) are necessary.^[^ ^6^ ^]^ Platinum nanoparticles have been considered as the most energetically efficient catalytic materials, but their use in large‐scale industrial setting is limited by the high cost and scarcity.^[^ ^7^ ^]^ Although tremendous efforts have been devoted toward the development of low‐cost HER catalysts based on earth‐abundant materials, their electrochemical performances are typically much lower than platinum.^[^ ^8^ ^]^

Another strategy to achieve cost‐effective water splitting cells is to increase the surface‐to‐volume ratio of the platinum catalysts via size reduction because only the surface atoms can actively participate in the catalytic reaction.^[^ ^9^ ^]^ In particular, downsizing the platinum to the single‐atom limit allows for nearly 100% utilization of the atoms for HER.^[9a,9b]^ To this end, recent studies have focused on creating isolated platinum atoms using metal oxides, carbon‐based materials, or 2D nanomaterials as a solid support.[^9^, ^10^] Atom‐scale defects on these support materials such as oxygen vacancies, unsaturated coordination sites, and heteroatom dopants serve as anchoring sites on which platinum atoms can bind to. Although the single‐atom platinum catalysts allow for significant enhancement in the catalytic activity compared to the platinum nanoparticles on a per atom basis,^[9a]^ their practical application in electrochemical water splitting is hindered because 1) scalable production of finely distributed defect sites at high densities is still challenging;^[^ ^11^ ^]^ 2) the catalyst loading needs to be kept low (typically < 1 wt%) to ensure atomic dispersion of platinum without nanoparticle formation, which often leads to a low electrochemical activity per unit electrode area;^[^ ^12^ ^]^ and 3) platinum atoms tend to detach from the anchoring sites and aggregate into nanoparticles during catalytic reaction.^[^ ^11^, ^13^ ^]^

Besides downsizing of the platinum catalysts, engineering of nanoscale particle morphology has also been intensively pursued to enhance the surface‐to‐volume ratio and the catalytic properties.^[6a]^ For example, platinum nanoparticles with nanobranches or flower‐like nanostructures offer more active sites compared to the platinum nanospheres conventionally used for hydrogen evolution, thereby achieving higher electrochemical performance for the same mass loading.^[^ ^14^ ^]^ Furthermore, the nanostructured catalytic surface promotes release of hydrogen bubbles from the electrodes compared to the flat counterpart, which is beneficial for achieving high production yield and operational stability especially under high current densities.^[^ ^15^ ^]^ Conventional approaches to produce nanostructured platinum catalysts were based on solution‐phase synthesis in homogenous liquid environments, which require long reaction times (tens of hours) because the reduction rate should be kept extremely slow to achieve kinetic control and induce anisotropic particle growth; otherwise, spherical nanoparticles form because they are thermodynamically favored.[^14^, ^16^] Moreover, the resulting nanocatalysts must be loaded onto an electrode using polymeric binders and therefore the electrochemically active surface area cannot be fully accessed during HER.^[^ ^17^ ^]^ Direct electrochemical synthesis of platinum nanoparticles on the electrode surface can drastically shorten the reaction time because of reduced energy barrier for nucleation at the solid/liquid interface and also circumvent the need for postprocessing steps involving binders.^[^ ^18^ ^]^ In particular, carbon‐based low‐dimensional nanomaterials such as carbon nanotubes, reduced graphene oxide, and graphene have been extensively investigated as a growth template for the electrochemical synthesis of platinum nanocatalysts.^[^ ^19^ ^]^ These materials, however, could only produce nanospheres or spiky nanoparticles with limited control over branch morphologies, which motivates an alternative materials platform that allows for the growth of more sophisticated nanostructures needed for enhancing the HER activity of Pt nanocatalysts.

Herein, we report a scalable electrochemical method to produce flower‐inspired platinum nanocatalysts (Pt nanoflowers [Pt NFs]) with well‐defined petal structures. Solution‐processed 2D molybdenum disulfide (MoS_2_) nanosheets were assembled into a semiconducting film onto a carbon paper (CP) substrate and served as a growth template to synthesize Pt NFs via electrochemical reduction of Pt precursor solution. Such flower‐shaped Pt nanocatalysts could not be realized with other templates we tested including the bare CP, metallic graphene, and insulating hexagonal boron nitride films, which highlights the uniqueness of our 2D semiconductor template. The HER electrodes decorated with Pt NFs exhibited lower overpotential and Tafel slope compared to those with spherical or spiky Pt nanocatalysts because of higher active surface area as well as improved operational stability resulting from facile release of hydrogen bubbles before coalescence. Using a reactive ion etching process, we could introduce additional sulfur vacancies to the MoS_2_ template and induce partial oxidation of MoS_2_ into metallic molybdenum oxide. This posttreatment enabled high‐density, uniform growth of Pt NFs, which further enhanced the HER performance to achieve more than a tenfold increase in the mass activity (25.8 A mg_Pt_^−1^) compared to the conventional Pt/C electrode (2.2 A mg_Pt_^−1^) at an overpotential of 0.1 V. The Pt NFs could be repeatedly synthesized over large areas from a single precursor solution (more than 30 times) with nearly identical particle morphology, areal density, and HER activities, thereby demonstrating the cost‐efficiency and scalability of our 2D semiconductor template‐assisted strategy.

## 2 Results and Discussion

### 2.1 Template‐Assisted Synthesis of Pt Nanoparticles

**Figure 1:**
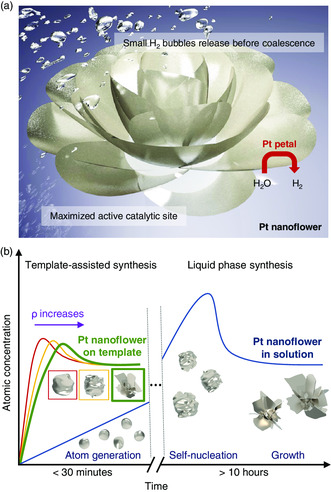
Template‐assisted synthesis of Pt NFs for HER. a) Schematic illustration of the Pt NF structure for increased active sites and efficient hydrogen bubble removal. b) La Mer diagrams describing mechanisms of nucleation and subsequent growth of Pt NFs for different synthesis methods.

The Pt NFs that we synthesized using our 2D semiconductor template are schematically illustrated in **Figure** **1**a. The Pt NF consists of multiple petals that significantly increase the surface area compared to a spherical nanoparticle of similar volume.^[^ ^20^ ^]^ This nanoscale morphology is greatly advantageous for maximizing the active sites available for catalyzing HER. In addition, the flower‐like nanoarchitecture can decrease the adhesion force between as‐formed H_2_ bubbles and the Pt surface.^[15b]^ The reduced adhesion promotes release of bubbles before they coalescence into larger ones, which enhances the catalytic activity and long‐term stability of the electrode.[^15^, ^21^] Figure 1b describes the advantage of our template‐assisted electrochemical synthesis strategy based on the LaMer model.[^18^, ^22^] Conventionally, Pt nanoparticles have been synthesized by chemically reducing a Pt precursor solution (e.g., H_2_PtCl_6_ or [Pt(NH_3_)_4_]Cl_2_) in a homogenous liquid environment (Figure 1b, diagram on the right). In the early stage, Pt atoms (Pt^0^) are generated from reduction of Pt ions with the presence of reductive solvents or reducing reagents. As the concentration of Pt atoms increases to a critical value above the supersaturation condition, the atoms can rapidly condense to form nuclei by overcoming the thermodynamic energy barrier for creating a solid phase. Such burst nucleation is accompanied by a sudden drop of Pt atom concentration below the critical value for new nucleation and further reduction of Pt ions contributes mostly to the growth of existing nuclei. This liquid phase synthesis based on homogeneous nucleation is limited as a general strategy to produce Pt NFs because the Pt reduction rate should be typically kept very low to promote anisotropic particle growth, which leads to a long reaction time (tens of hours) for the overall nucleation and growth stages.[^14^, ^16^] To mitigate this challenge, we introduce 2D material films as a growth template to synthesize Pt nanoparticles via electrochemical deposition (Figure 1b, diagram on the left). Because the energetic barrier for Pt nucleation is drastically lowered by the 2D material surface,^[18a]^ synthesis time can be dramatically reduced. Furthermore, the growth mechanisms can be engineered to control the particle morphology by altering the reaction kinetics of Pt species based on current modulation.^[^ ^23^ ^]^

**Figure 2:**
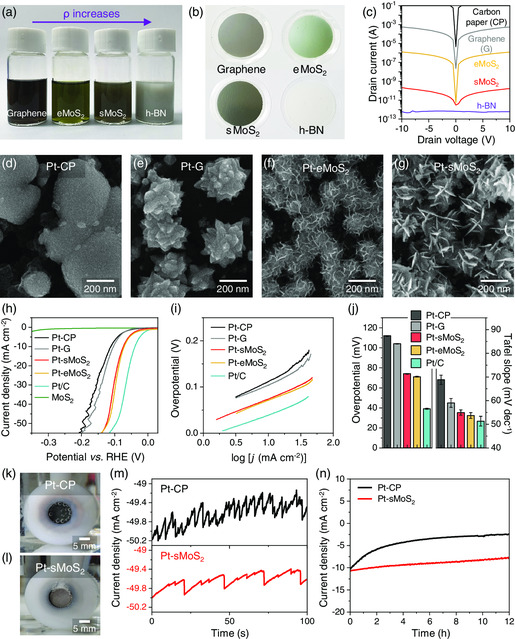
2D nanomaterial templates for controlled Pt nanoparticle synthesis. a,b) Photographs of graphene, eMoS_2_, sMoS_2_, and h‐BN dispersions and the templates made from the dispersions. c) Semilogarithmic current−voltage curves of CP, graphene, eMoS_2_, sMoS_2_, and h‐BN templates. d–g) Top‐view SEM images of Pt nanoparticles grown on CP, graphene, eMoS_2_, and sMoS_2_ under 0.5 V. h) LSVs, i) Tafel plots, and j) overpotentials (*j* = 10 mA cm^−2^) and Tafel slopes of the template‐assisted Pt nanoparticles in a H_2_‐saturated 1 m KOH solution. k,l) Photographs of hydrogen bubbles generated from Pt‐CP and Pt‐sMoS_2_ films during HER. m,n) Corresponding chronoamperometry curves.

**Figure** **2**a shows dispersions of 2D nanosheets that were used to prepare the Pt growth template. Based on the expectation that the Pt particle morphology would greatly depend on the electronic properties of the template, we tested various 2D nanosheets including metallic graphene (G), semiconducting molybdenum disulfide (MoS_2_), and insulating hexagonal boron nitride (h‐BN). For MoS_2_, two types of nanosheets were prepared (Experimental Section): electrochemically exfoliated MoS_2_ with an average lateral size of ≈500 nm (eMoS_2_) and liquid‐phase‐exfoliated MoS_2_ with a smaller average lateral size of ≈110 nm (sMoS_2_). The dispersions were vacuum‐filtrated through anodic aluminum oxide (AAO) membranes to prepare thin films of 2D nanosheets (Figure 2b), which were released from the AAO after drying and then transferred onto CPs to fabricate electrodes. To compare the electrical properties of the 2D nanosheet films, we measured the current–voltage (*I*–*V*) curves in a two‐electrode configuration (Figure 2c). For all cases, the nanosheet film/CP composites showed lower current levels compared to the bare CP substrate. The current level for the graphene sample was the highest among the tested 2D materials, which indicates that the graphene template was the most conductive because of the metallic character of the nanosheets. Notably, the eMoS_2_ film exhibited approximately two orders of magnitude higher current level than the sMoS_2_ sample because the nanosheet network tends to be more conductive when the lateral size of nanosheets is larger.^[^ ^24^ ^]^ The h‐BN template was characterized by negligible current levels because the constituent nanosheets were insulating.

The electrodes were then loaded into a three‐electrode cell with a H_2_PtCl_6_ solution (Experimental Section), followed by application of a DC voltage of 0.5 V (vs RHE) for 10 min to synthesize Pt nanoparticles (Figure 2d–g). Interestingly, the morphology and areal density of Pt nanoparticles significantly varied depending on the growth template. Spherical Pt nananoparticles were formed on the bare CP substrate (Pt‐CP) with an average diameter of 340 nm (Figure 2d and S1, Supporting Information). The areal density of the Pt nanospheres (1.7 particles μm^−2^) was much lower than Pt nanoparticles grown on other templates because Pt nucleation was more difficult due to the chemical inertness of CP.^[^ ^25^ ^]^ When the graphene nanosheets were used as a template, the resulting Pt nanoparticles (Pt‐G) consisted of spherical cores and spike‐like protrusions (Figure 2e). Such spiky nanoparticles were smaller (average particle size: 300 nm) compared to the Pt‐CP case (Figure S1, Supporting Information). In addition, the areal density of the particles was also higher (4.5 particles μm^−2^), which is most likely because the graphene template had more anchoring sites that stabilized the formation of Pt particles such as edges of the nanosheets and structural defect in the basal plane.^[^ ^26^ ^]^ Notably, Pt nanoparticles grown on eMoS_2_ (Pt‐eMoS_2_) and sMoS_2_ (Pt‐sMoS_2_) were characterized by distinct flower shapes with sharp, petal‐like nanoflakes (Figure 2f,g). The formation of Pt NFs was further confirmed by elemental mapping based on energy‐dispersive X‐ray spectroscopy (EDS) (Figure S2, Supporting Information). The average particle sizes and areal densities were 130 nm and 43 particles μm^−2^ for Pt‐eMoS_2_ and 270 nm and 17 particles μm^−2^ for Pt‐sMoS_2_, respectively (Figure S1, Supporting Information). In the case of the h‐BN template, Pt nanoparticles were not formed because the surface was insulating and therefore electric charge required to reduce Pt ions could not be properly supplied (Figure S3, Supporting Information).

The template dependency of the Pt nanoparticle morphology can be understood based on the difference in reduction rates on each template. In the case of MoS_2_ template, we believe that the reduction of Pt ions was slightly faster than the diffusive transport of precursors such that a radial concentration gradient was developed around the Pt nuclei in the early stage of growth.^[^ ^23^, ^27^ ^]^ Under this condition, local protrusions of the particles such as edges and corners tend to grow faster as they are exposed to higher concentrations of precursors compared to the regions closer to the particle core.^[^ ^23^, ^27^ ^]^ As the electrodeposition proceeds, this preferential growth of protrusive features becomes more pronounced, which accounts for the formation of nanopetals on Pt‐eMoS_2_ and Pt‐sMoS_2_. Based on the transmission electron microscope (TEM) images of Pt NFs (Figure S4, Supporting Information), we found that the surface of nanopetals mainly showed nanoscale domains exhibiting lattice fringes with an interplanar spacing of ≈3.8 Å, which corresponded to the {100} planes of the face‐centered cubic Pt.^[^ ^28^ ^]^ This surface atomic structure suggests that the nanopetals predominantly consisted of (110)‐oriented grains most likely because the nanopetals grew in some preferred directions. The proposed growth mechanism is further supported by the chronoamperometry curve during the Pt growth process on the sMoS_2_ template (Figure S5, Supporting Information), where the current initially dropped and monotonically increased similar to the previous work on silver dendrite growth.[^23^, ^27^] Compared to the MoS_2_ films, graphene template can provide more current under the same potential and hence the rate of Pt reduction on the surface is faster while the diffusion coefficient for the transport of precursors is nearly constant.^[^ ^27^ ^]^ Accordingly, the precursor is depleted further away from each Pt nuclei, generating a concentration gradient that is more gradual compared to the case of MoS_2_. This growth environment could lead to the formation of nanospikes instead of well‐defined nanopetals. Further increase in conductivity of the template by using the bare CP would result in a condition where reduction rate is much faster compared to the diffusive transport and the concentration gradient barely develops around the Pt nuclei. This condition results in the Pt nanoparticles that are spherical.

### 2.2 Catalytic Activity of Pt NFs for HER

To verify whether the HER activity can be enhanced by engineering the morphology of Pt nanocatalysts, we compared the electrochemical properties of the electrodes decorated with template‐assisted Pt nanoparticles (Pt‐CP, Pt‐G, Pt‐sMoS_2_, and Pt‐eMoS_2_). Figure 2h shows linear sweep voltammograms (LSVs) of the electrodes during HER in 1 m KOH solution, where the measurement from the conventional Pt/C catalysts is also provided for comparison. The onset potential at which the current density began to rise was lower for Pt‐sMoS_2_ and Pt‐eMoS_2_ compared to Pt‐CP and Pt‐G. The decreased onset potential for Pt‐sMoS_2_ and Pt‐eMoS_2_ cases can be attributed to the larger surface area of Pt NFs that are active for HER than those of Pt nanospheres (Pt‐CP) and spiky Pt nanoparticles (Pt‐G) as well as higher areal density of Pt NFs because the MoS_2_ layer only had negligible contribution to the current density within the tested potential range (Figure 2h, MoS_2_ and S1, Supporting Information). The catalytic activity of the electrodes was further investigated by analyzing the Tafel plot constructed from the LSV data (Figure 2i). In particular, overpotentials (*η*) to achieve a current density (*j*) of 10 mA cm^−2^, which is widely used as a metric to describe the HER performance, were 74 and 71 mV for Pt‐sMoS_2_ and Pt‐eMoS_2_, respectively, while Pt‐CP and Pt‐G exhibited higher *η* values of 112 and 104 mV. In addition, the Tafel slopes were 69, 59, 55, and 53 mV dec^−1^ for the Pt‐CP, Pt‐G, Pt‐sMoS_2_, and Pt‐eMoS_2_, respectively (Figure 2j). Based on the comparison of *η* values and Tafel slopes, we confirmed that Pt NFs (Pt‐sMoS_2_, and Pt‐eMoS_2_) exhibited higher HER activities compared to spiky Pt nanoparticles (Pt‐G), all of which performed better than Pt nanospheres (Pt‐CP). Interestingly, Pt‐eMoS_2_ was slightly more active than Pt‐sMoS_2_, with lower *η* and Tafel slope because the particle size of NFs was smaller and the areal density was higher. The benefit of NF structure was further highlighted by the hydrogen evolution behavior under high current densities. Different from the Pt‐CP and Pt‐G cases where LSV curves were characterized by noticeable fluctuations when the current density was higher than 40 mA cm^−2^, Pt‐sMoS_2_ and Pt‐eMoS_2_ samples showed smooth LSV curves without such characteristic features (Figure 2h). To clarify the role of the flower‐like structure in improving the operational stability during water splitting, we performed a side‐by‐side comparison of hydrogen evolution behaviors on Pt‐CP and Pt‐sMoS_2_ at a fixed overpotential (target current density: 50 mA cm^−2^). In the case of Pt‐CP, hydrogen bubbles could not be easily detached from the surface and blocked the KOH solution from Pt nanocatalysts until they coalesced into larger ones such that the buoyant force could overcome the gas/solid interfacial energy (Figure 2k and Video S1, Supporting Information). In contrast, the surface of Pt‐sMoS_2_ was aerophobic, thereby promoting the release of hydrogen bubbles before their coalescence to allow for faster recovery of the catalytic surface compared to Pt‐CP (Figure 2l and Video S2, Supporting Information). As a result, Pt‐sMoS_2_ did not show current fluctuations in the chronoamperometry curve (Figure 2m), which were clearly observed in the case of Pt‐CP (note that the periodic decrease in current density followed by a sharp increase at 25 s intervals was caused by bubbles trapped by the holder surrounding the electrode, which was not related to the intrinsic properties of the catalytic surface). Notably, Pt‐sMoS_2_ maintained an efficiency of about 80% even after 12 h of HER reaction at a current density of 10 mA cm^−2^ despite the absence of binders that are necessary to achieve the long‐term stability (Figure 2m). In contrast, Pt‐CP showed more than 70% decrease in the current density after 12 h, which clarifies the merit of introducing NF structures for reducing the bubble adhesion.

**Figure 3:**
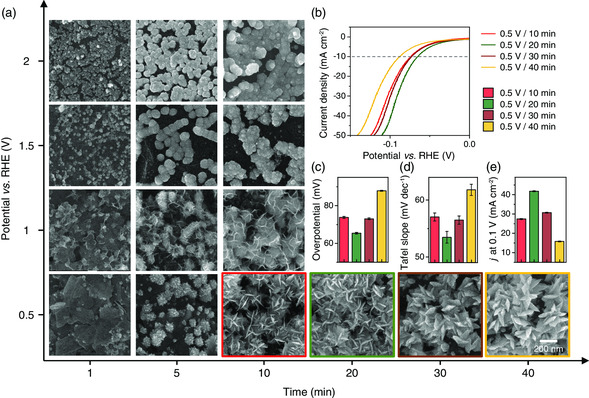
Effect of applied voltage and reaction time on morphology of Pt nanoparticles. a) SEM images of Pt nanoparticles grown on sMoS_2_ under different potentials for different times. b–e) Linear sweep voltammograms, overpotentials (*j* = 10 mA cm^−2^), Tafel slopes, and current densities (*η* = 0.1 V) of Pt NFs formed with different growth times under 0.5 V. HER was performed in a H_2_ saturated 1 m KOH solution.

Based on the sMoS_2_ template, we further investigated tunability in the nanoparticle morphology by performing electrodeposition of Pt under different potentials for different times (**Figure** **3**a). Similar to the case with 0.5 V, Pt growth under 1 V produced NFs but their petal structures were less pronounced. As the electrodeposition potential increased to 1.5 V, we observed Pt nanospheres in the early stage of growth up to 5 min, after which they grow into spiky nanoparticles. Further increase in potential to 2 V resulted in Pt nanospheres. We note that the relation between the deposition potential and particle morphology on sMoS_2_ was similar to the trend observed from particles synthesized on different templates (Figure 2d–g). Under low potentials, the current level is low such that the reduction rate is sufficiently low to develop concentration gradient around the nuclei needed for nanopetal formation. When higher potentials were applied (higher current levels), the concentration gradient becomes less pronounced and the resulting Pt nanoparticles are more spherical. Thus, the MoS_2_ template offers a distinct advantage compared to other 2D templates we tested because Pt nanoparticles with a variety of shapes could be synthesized on the single material platform by simply changing the electrodeposition potential and growth time.

The Pt NFs that were formed under 0.5 V for different times were characterized in more detail to identify an optimal growth condition for enhancing the HER performance. Up to 5 min, the nanoparticles consisted of spikes rather than fully developed petal structures. As the growth time increased to 10 min, the particles evolved into NFs with sharp petals while the overall particle size also increased. For prolonged reduction of Pt (20, 30, and 40 min), both the particle size and the thickness of petals became larger. Interestingly, NFs started to merge with each other to develop into film‐like deposits when the growth time was longer than 20 min. The difference in particle morphology resulted in a marked difference in catalytic activities. Figure 3b presents LSV curves of Pt‐sMoS_2_ electrodes resulting from electrodeposition at 0.5 V for different times. Significantly, Pt NFs formed with 20 min growth showed the best HER performance with the lowest *η* at *j* = 10 mA cm^−2^ (65 mV) and Tafel slope (53 mV dec^−1^) and the highest *j* at *η* = 0.1 V (42 mA cm^−2^) (Figure 3c–e). The decreased performance of electrodes corresponding to 30 and 40 min Pt growth was attributed to the reduction in available surface area, although the Pt mass loading was higher compared to the case of 20 min deposition. The results highlight that not only the mass loading but also the well‐defined nanopetal structures are critical for boosting the HER activities.

### 2.3 Surface Modification of MoS_2_ for Increasing Density of Pt NFs

**Figure 4:**
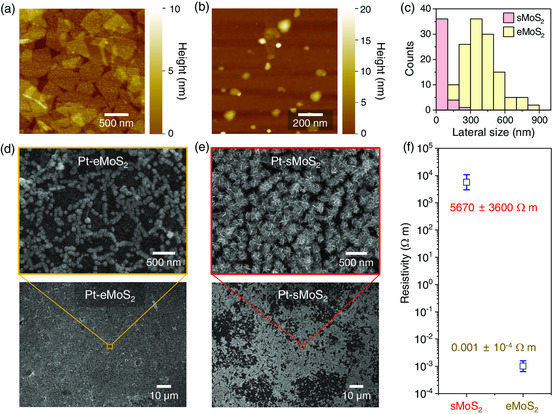
Effect of MoS_2_ nanosheet dimensions on Pt nanoparticle growth. AFM images of a) eMoS_2_ and b) sMoS_2_ nanosheets. c) Histogram summarizing the lateral size distributions of the nanosheets. SEM images of Pt NFs grown on d) eMoS_2_ and e) sMoS_2_ templates. f) Diagram showing the average and standard deviation of resistivity of the templates.

To clarify the reason why the size and areal density of Pt NFs were different depending on the type of MoS_2_ template and identify an approach to improve the HER performance further, we conducted a more detailed study on the relation between the characteristics of MoS_2_ nanosheet films and the resulting Pt NF morphology. **Figure** **4**a,b shows AFM images of eMoS_2_ and sMoS_2_ nanosheets, respectively. The lateral sizes of eMoS_2_ ranged from 100 to 900 nm, which was much larger than sMoS_2_ (<300 nm) with the lateral sizes mostly under 100 nm (Figure 4c). As a result, eMoS_2_ nanosheets could form a film with better intersheet connectivity and thickness uniformity compared to sMoS_2_ nanosheets. The particle morphology and spatial distribution of Pt NFs grown on eMoS_2_ and sMoS_2_ templates were then compared side‐by‐side based on SEM images obtained at different magnifications (Figure 4d,e). Pt NFs on sMoS_2_ exhibited a larger size and more distinct petal structures compared to those on eMoS_2_. For both cases, Pt NFs were created mostly at the edges of MoS_2_ nanosheets because the edge sites are active for Pt nucleation due to the presence of undercoordinated Mo atoms while the basal plane is mostly inactive.^[^ ^29^ ^]^ Although the sMoS_2_ template offered significantly higher density of edge sites compared to the eMoS_2_ counterpart as the lateral size of nanosheets was much smaller, the areal density of NFs was lower for the sMoS_2_ case. The reason is attributed to the spatial uniformity of electrical properties of each template (Figure 4f): the sMoS_2_ film showed a larger average resistivity and spatial heterogeneity compared to the eMoS_2_ sample, which could lead to nonuniform current distribution during Pt NFs growth and create microscale regions that were not coated with Pt NFs. As a result, the Pt‐sMoS_2_ showed lower catalytic activities compared to Pt‐eMoS_2_, although a large portion of the sMoS_2_ surface was more densely coated with Pt NFs than the eMoS_2_ template.

**Figure 5:**
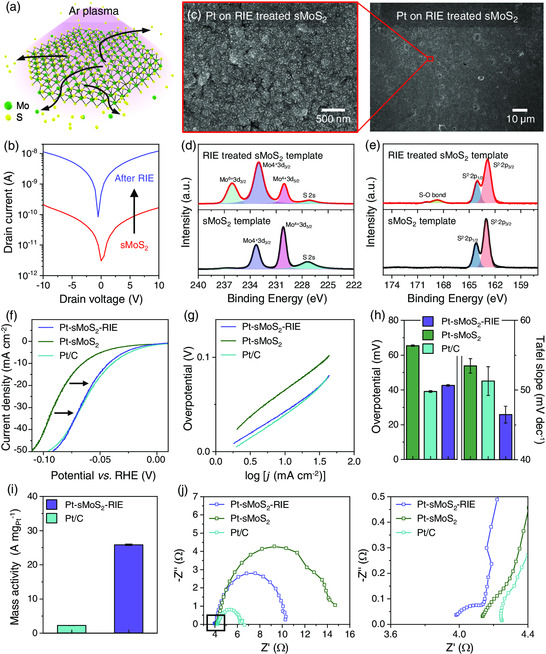
High‐density, uniform growth of Pt NFs via surface modification of sMoS_2_. a) Schematic illustration of the Ar RIE treatment for generating sulfur vacancies and MoO_*x*_ on the sMoS_2_ nanosheet film. b) Semilogarithmic current−voltage characteristics of pristine and RIE‐treated sMoS_2_ templates. c) SEM images of Pt NFs deposited on RIE‐treated sMoS_2_ template. d,e) XPS analysis of pristine and RIE‐treated sMoS_2_ templates. f) LSVs, g) Tafel plots, and h) overpotentials (*j* = 10 mA cm^−2^) and Tafel slopes of the Pt particles on each templates in a H_2_‐saturated 1 M KOH solution. i) Mass activities of Pt‐sMoS_2_‐RIE and commercial Pt/C electrodes at *η* = 0.1 V. j) Nyquist plots comparing charge transfer resistances and ohmic resistances of Pt‐sMoS_2_‐RIE, Pt‐sMoS_2_, and commercial Pt/C electrodes.

The observation that higher density of defect sites and spatial uniformity of electrical properties are beneficial for increasing the density of Pt NFs motivates the surface modification of sMoS_2_ film for improving our template‐assisted Pt NF synthesis. To this end, we treated the sample with reactive ion etching (RIE) using Ar plasma (**Figure** **5**a) because 1) such process was expected to generate sulfur vacancies on the basal plane that promote growth of Pt atoms^[^ ^29^, ^30^ ^]^; and 2) heterogeneity in resistivity would be mitigated because MoS_2_ would partially convert to metallic molybdenum oxide (MoO_*x*_) upon exposure of the sulfur vacancies to the atmosphere and conductivity of the template would increase.^[^ ^31^ ^]^ Furthermore, the MoO_*x*_ can also serve as an active material to reduce Pt ions based on a previous study.^[^ ^32^ ^]^ Consistent with our hypothesis, Ar RIE treatment of sMoS_2_ template resulted in approximately two orders of magnitude higher current levels compared to the pristine sample (Figure 5b). Significantly, Pt nuclei could form much more densely on the RIE‐treated sMoS_2_ surface (Figure S6, Supporting Information), which enabled high‐density, uniform growth of Pt NFs throughout the template (Figure 5c). The elemental composition and chemical bonding states of RIE‐treated sMoS_2_ were characterized by X‐ray photoelectron spectroscopy (XPS) to corroborate our observations. The decrease in S/Mo ratio from 1.86 to 0.54 reveals that sulfur vacancies were created by treating the surface with Ar RIE. In addition, the prominent Mo^6+^ 3d_3/2_ and S–O peaks in the XPS spectra for the RIE‐processed sMoS_2_ template confirmed that the improved conductivity resulted from the formation of MoO_*x*_ (Figure 5d,e). We note that the RIE‐assisted Pt NF synthesis strategy could only be applied to sMoS_2_ because the eMoS_2_ treated with RIE could only form Pt nanospheres, although the particle density was significantly increased (Figure S7, Supporting Information).

We confirmed that the HER activities of the electrode were enhanced when Pt NFs were synthesized at high density using the RIE‐treated sMoS_2_ template (Pt‐sMoS_2_‐RIE) (Figure 5f–h). Pt‐sMoS_2_‐RIE showed reduced *η* at 10 mA cm^−2^ and Tafel slope (43 mV and 46 mV dec^−1^) compared to Pt‐sMoS_2_ that were grown for 20 min (65 mV and 53 mV dec^−1^) and even achieved lower Tafel slope than commercial Pt/C nanocatalysts (51 mV dec^−1^). Based on the mass loading of MoS_2_ (0.005 mg, Experimental Section) and the mass ratio of MoS_2_ and Pt (7:3) determined from the TEM EDS energy spectra (Figure S8, Supporting Information), we found that the Pt loading for the Pt‐sMoS_2_‐RIE was approximately 0.002 mg, which was less than 10% of the loading on the Pt/C electrode (0.023 mg). As a result, the Pt‐sMoS_2_‐RIE achieved a dramatic increase (more than tenfold) in the mass activity (25.8 A mg_Pt_^−1^) compared to the Pt/C electrode (2.2 A mg_Pt_^−1^) at *η* = 0.1 V (Figure 5i). Furthermore, the Nyquist plot indicated that the Ohmic resistance of Pt‐sMoS_2_‐RIE was the lower than Pt/C because polymeric binders were not used in the fabrication process (Figure 5j).

### 2.4 Cost Efficiency and Scalability of Template‐Assisted Pt NF Synthesis

**Figure 6:**
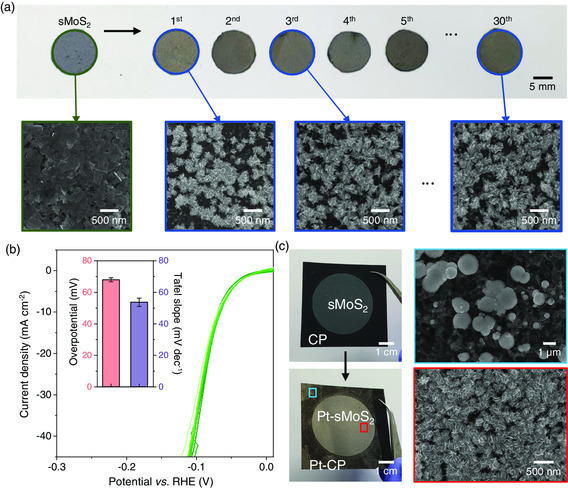
Recyclability of growth solution for cost‐efficient, scalable synthesis of Pt NFs. a) Photographs and SEM images of Pt NFs on sMoS_2_ films for different growth cycles. b) LSVs, overpotentials (*j* = 10 mA cm^−2^) and Tafel slopes of Pt NFs for different growth cycles. c) Large‐area synthesis of Pt NFs (4 × 4 cm^2^).

The merit of our process is that Pt NFs can be repeatedly synthesized from the same growth solution (≈$6 for 50 mL) for nearly unlimited times. This aspect offers a distinct advantage over the conventional Pt nanocatalysts because a certain volume of Pt/C ink is bound to produce limited number of HER electrodes. **Figure** **6**a shows photographs and SEM images of the Pt NFs formed on independent sMoS_2_ templates up to 30 iterations of Pt growth. The surface density and particle morphology of the Pt NFs were nearly identical throughout the repeated electrodeposition processes. As a result, HER catalytic activities of the electrodes were nearly identical based on the LSV curves, overpotential, and Tafel slope values (Figure 6b). To highlight the scalability of our strategy, we also demonstrate fabrication of Pt‐sMoS_2_ samples over large areas (>10 cm^2^). As can be observed from SEM images, Pt NFs could only form on the regions coated with the sMoS_2_ template, while spherical particles are created on the bare CP surface not covered by sMoS_2_ (Figure 6c).

## 3 Conclusion

In summary, we have demonstrated a facile strategy for scalable production of flower‐inspired Pt nanocatalysts by using semiconducting MoS_2_ nanosheet films as a growth template for electrodeposition. The Pt NFs with well‐defined petal structures achieved improved HER performance compared to spherical or spiky Pt nanocatalysts as well as operational stability benefiting from the sophisticated nanoscale morphology. The MoS_2_ template offered flexibility in designing the shape and areal density of Pt nanoparticles, which could be easily controlled by changing the electrodeposition potentials or applying a posttreatment with plasma. This level of tunability was not possible in other 2D material systems tested and important for the synthesis of HER electrodes that performed better than the state‐of‐the‐art Pt/C platform. Significantly, the template‐assisted NF growth allowed for nearly unlimited iterations of electrode fabrication from a single batch of precursor solution over large areas (>10 cm^2^), which highlighted the cost‐efficiency of our strategy. We speculate that our work will not only serve as an approach to take a step forward to achieve carbon neutrality but also drive advances in other research areas such as plasmonics, environmental remediations, and chemical sensors, where nanoscale engineering of nanoparticle morphology is crucial.

## 4 Experimental Section

4.1

4.1.1

##### Exfoliation of 2D Materials

To prepare graphene nanosheets, graphite (Sigma‐Aldrich) was electrochemically exfoliated in *N*,*N*‐dimethylformamide (Sigma‐Aldrich) and centrifuged at 4500 rpm for 30 min to remove unexfoliated powders. sMoS_2_ nanosheets were produced by exfoliating MoS_2_ powders (Sigma‐Aldrich) using a tip sonicator operated at 40 W for 2 h in a mixture of ethanol and deionized (DI) water (2:1 volume ratio) surrounded by an ice bath. The as‐prepared dispersion was centrifuged at 7500 rpm for 1 h to discard the unexfoliated powders. To obtain eMoS_2_ nanosheets, electrochemical exfoliation of MoS_2_ crystals (HQ graphene) was performed using tetraheptylammonium intercalants based on literature precedent.^[^ ^33^ ^]^ The resulting nanosheets were dispersed in the ethanol/DI water mixture. Boron nitride (h‐BN) nanosheets were obtained by applying a mild ultrasonication process to h‐BN powders (Sigma‐Aldrich) in isopropanol.

##### 2D Material Template Fabrication

For 2D material film formation, the dispersions containing graphene, eMoS_2_, and h‐BN nanosheets were vacuum‐filtrated through an AAO membrane with 25 nm pores (Whatman Anodisc) at a rate of 1 mL min^−1^. To produce sMoS_2_ films, graphene nanosheet film was first coated on the AAO membrane prior to the vacuum filtration of sMoS_2_ dispersion to prevent the nanosheets from penetrating through the pores.^[^ ^34^ ^]^ The mass loading of the sMoS_2_ (0.005 mg cm^−2^) was determined by multiplying the volume of the dispersion and the concentration, which was obtained from the absorbance spectra (Figure S9, Supporting Information) and the literature extinction coefficient (*α*_672_ = 3400 mL mg^−1^ m^−1^).^[^ ^34^, ^35^ ^]^ The resulting 2D nanosheet films were dried in a vacuum oven at 60 °C overnight after a soft baking on a hot plate at 70 °C for 5 min. Then, the dried template was separated from AAO and transferred to a CP (Freudenberg H23C2, Fuel Cell Store). RIE‐treated sMoS_2_ and eMoS_2_ templates were prepared by applying Ar plasma at 15 W for 3 min.

##### Synthesis of Platinum Nanoparticles via Electrodeposition

The electrochemical synthesis of Pt nanoparticles was performed in an alkaline precursor solution prepared by adding H_2_PtCl_6_ (8 wt% solution, Sigma‐Aldrich) to a 20:1 mixture of 0.5 m NaCl and 0.1 m KOH. The concentration of the Pt precursor was 2.8 mm. A graphite rod (Sigma‐Aldrich, 99.9995%) and an Ag/AgCl/KCl (saturated) electrode were used as the counter and reference electrodes, respectively. The potentiostat was used to apply a potential of 0.5–2 V (vs RHE) for a desired duration at room temperature.

##### Materials Characterization

The morphology of the samples was characterized using a field‐emission scanning electron microscope (JEOL JSM‐7000F). The thickness and lateral size of the MoS_2_ nanosheets were determined by using an atomic force microscope (Park Systems NX10). For XPS analysis, an ultrahigh vacuum (UHV) Thermo Fisher Scientific K‐Alpha+ XPS spectrometer equipped with a monochromated Al Kα X‐ray source (≈200 μm spot size) was used for collecting the data. For *I*–*V* measurements, 3 nm of Cr and 50 nm of Au were deposited via thermal evaporation through a shadow mask to form electrodes (width: 450 μm, separation: 100 μm). The samples were characterized under vacuum (≈10^−5^ Torr) using a vacuum probe station with a Keithley SCS‐4200 system at room temperature.

##### Electrochemical Measurements

Electrochemical measurements were performed using a standard three‐electrode cell controlled by a potentiostat. A graphite rod and an Ag/AgCl/KCl (saturated) electrode were used as the counter and reference electrodes (*E* (RHE) = *E*(Ag/AgCl) + 0.0591 × pH + 0.197 V in 1 m KOH, where *E* denotes the potential), respectively. Linear sweep voltammetry measurements were conducted at a scan rate of 2 mV s^−1^. Each data point and error bar in the plots represent the average and standard deviation from three to four measurements. Electrochemical impedance spectroscopy measurements were performed in a frequency range of 0.01 − 100 000 Hz at 3 mA cm^−2^ (vs RHE) to determine the series resistance (*R*_s_) and charge transfer resistance (*R*_ct_). The electrode coated with Pt/C catalysts (Sigma‐Aldrich) was also characterized to compare the electrochemical performance of our Pt nanoparticles with the commercial HER catalyst. We applied the Pt/C ink on the electrode (5 mg of 20 wt% Pt/C powder mixed with 400 μL of isopropanol and 30 μL of 5% Nafion) to yield a Pt loading of 0.023 mg cm^−2^.

## Conflict of Interest

The authors declare no conflict of interest.

## Supporting information

Supplementary Material

## Data Availability

The data that support the findings of this study are available from the corresponding author upon reasonable request.
