# Supplementary material for: Scalable Synthesis of Pt Nanoflowers on Solution‐Processed MoS2 Thin Film for Efficient Hydrogen Evolution Reaction
Source: Small Sci. 2022 Aug 2;2(9):2200043. doi: 10.1002/smsc.202200043 (PMC11936019; doi:10.1002/smsc.202200043)
Supplement: Supplementary file 1 — Supplementary Material [file SMSC-2-2200043-s001.zip › smsc202200043-sup-0001-SuppData-S1.docx]

Supporting Information

Scalable Synthesis of Pt Nanoflowers on Solution-Processed MoS_2_ Thin Film for Efficient Hydrogen Evolution Reaction

Yun Seong Cho,^a,^ ^‡^ Dongjoon Rhee,^a,‡^ Jeongha Eom,^a^ Jihyun Kim,^a^ Myeongjin Jung,^a^ Youngdoo Son,^c^ Young-Kyu Han,^d^ Ki Kang Kim,^e,f^ and Joohoon Kang ^a,b,^*

^a^ School of Advanced Materials Science and Engineering, Sungkyunkwan University (SKKU), Suwon 16419, Republic of Korea

^b^ KIST-SKKU Carbon-Neutral Research Center, Sungkyunkwan University (SKKU), Suwon 16419, Republic of Korea

^c^ Department of Industrial and Systems Engineering, Dongguk University-Seoul, Seoul 04620, Republic of Korea

^d^ Department of Energy and Materials Engineering, Dongguk University-Seoul, Seoul 04620, Republic of Korea

^e^ Department of Energy Science, Sungkyunkwan University (SKKU), Suwon 16419, Republic of Korea

^f^ Center for Integrated Nanostructure Physics (CINAP), Institute for Basic Science (IBS), Sungkyunkwan University (SKKU), Suwon 16419, Republic of Korea

^‡^ These authors contributed equally to this work.

* Corresponding author: [joohoon@skku.edu](mailto:joohoon@skku.edu)


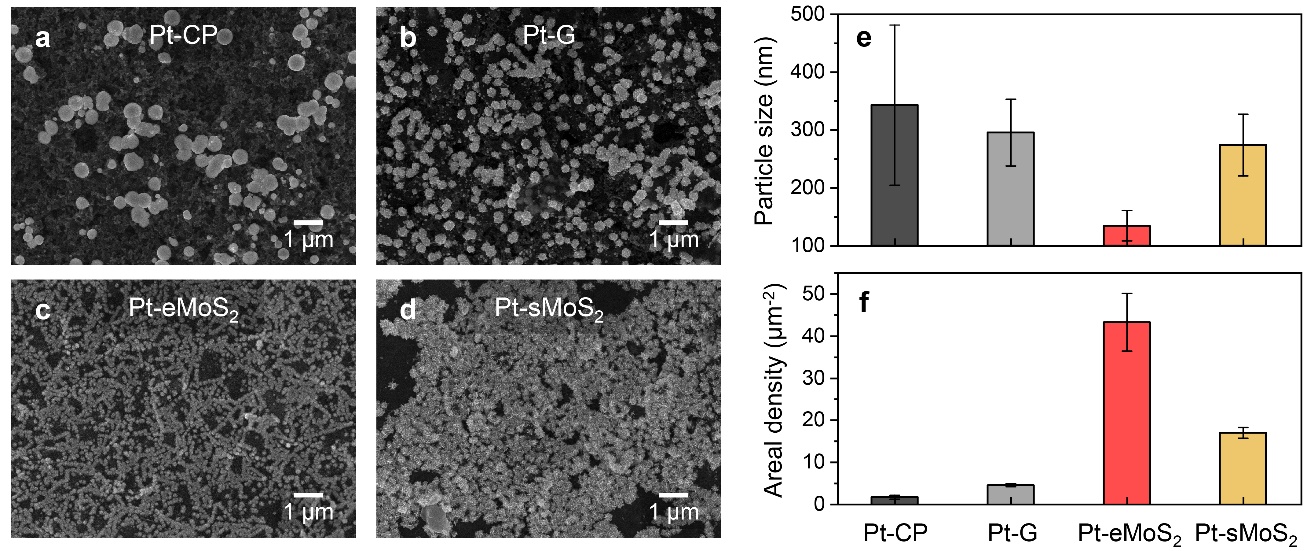


**Figure S1. Morphology and spatial distribution of Pt nanoparticles. (a–d)** Pt nanoparticles on different 2D templates. **(e**,**f)** Overall size and areal density of the Pt nanoparticles.


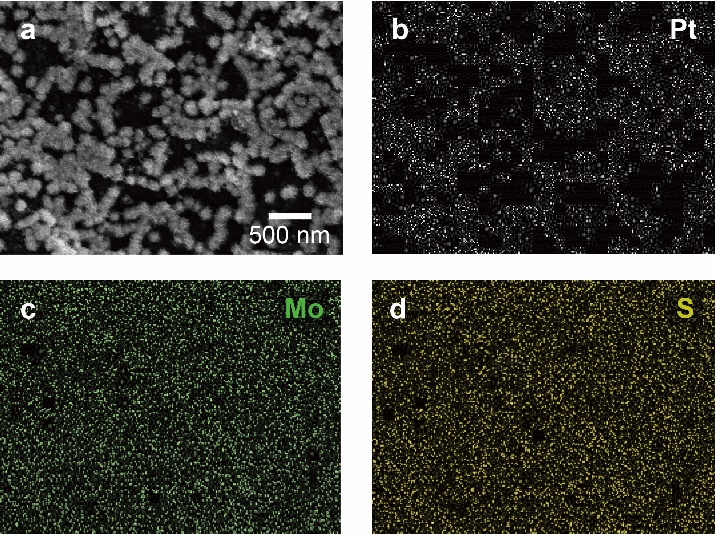


**Figure S2. Elemental mapping of the Pt-eMoS_2_ obtained from EDS. (a)** SEM image of the Pt nanoparticles grown on eMoS_2_. (**b**,**c**,**d**) Spatial mapping of Pt, Mo, and S elements on the same area imaged by SEM.


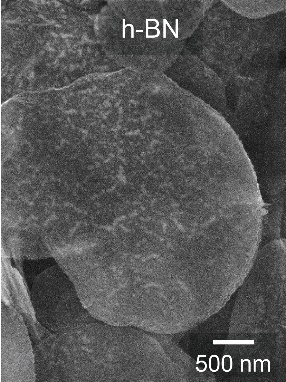


**Figure S3. Top-view SEM image of h-BN after the electrochemical Pt growth process.** No Pt nanoparticles were observed suggesting that h-BN cannot be used as a growth template**.**


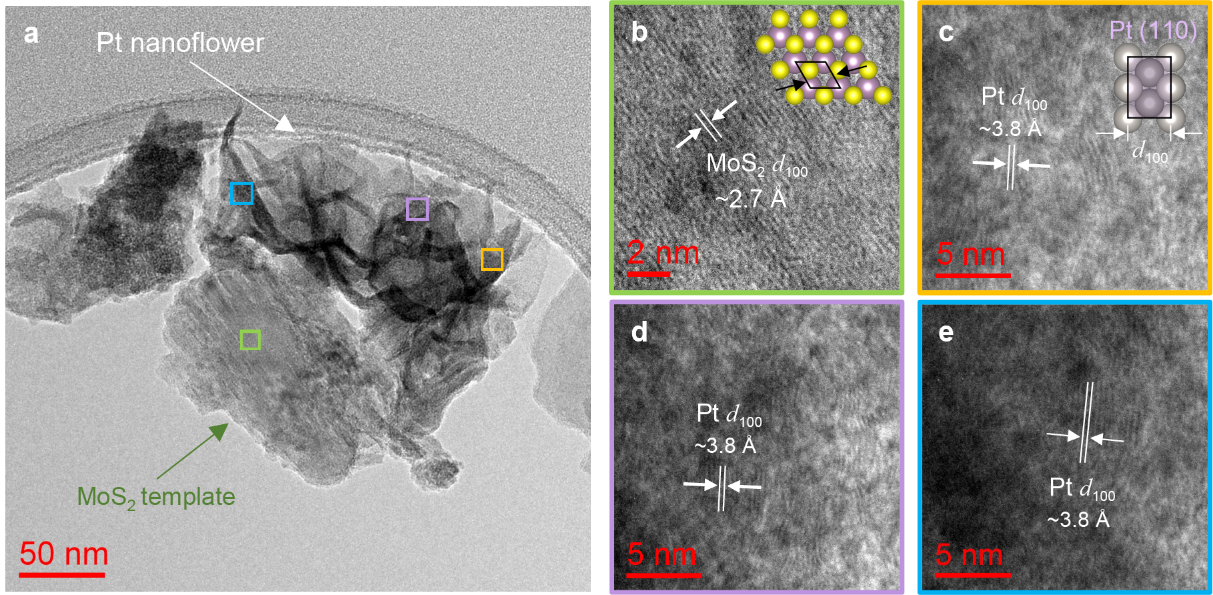


**Figure S4. Morphology and crystal structure of Pt nanoflowers on sMoS_2_** **(a)** TEM image of the Pt-sMoS_2_. **(b)** HRTEM image of the sMoS_2_ template. **(c**–**e)** HRTEM images of the nanoflowers in the petal regions.


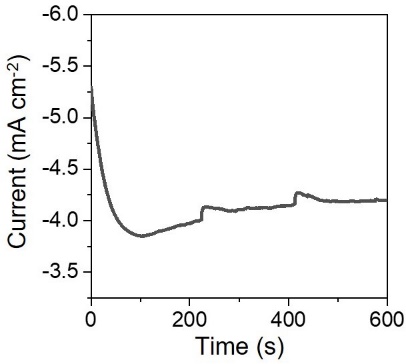


**Figure S5. Chronoamperometry curve during the Pt nucleation and growth process on sMoS_2_**.


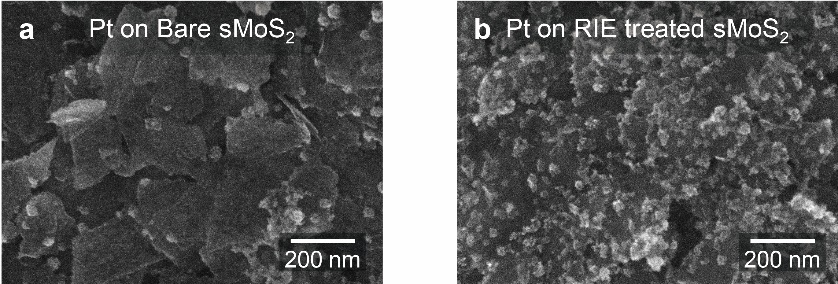


**Figure S6. Effect of RIE treatment on Pt nucleation.** Top-view SEM images of Pt nanoparticles nucleated on **(a)** bare sMoS_2_ and **(b)** RIE-treated sMoS_2_.


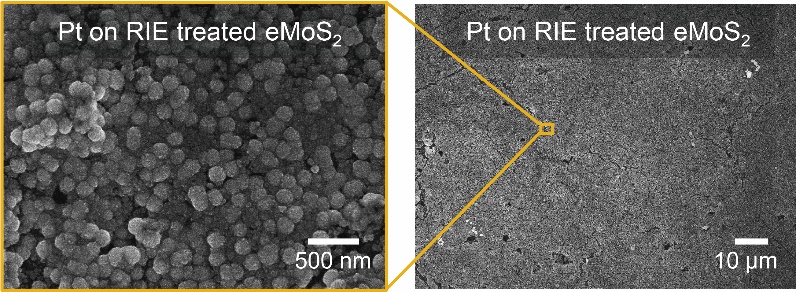


**Figure S7. SEM images of Pt nanoparticles deposited on RIE-treated eMoS_2_ template.**


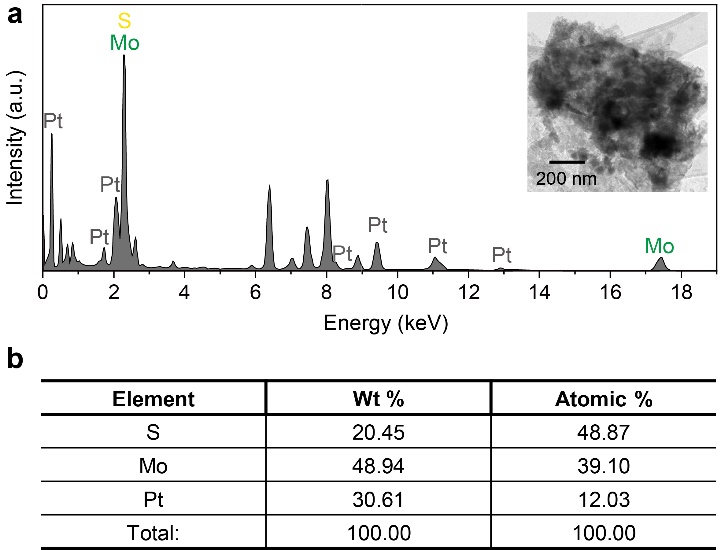


**Figure S8. Elemental analysis of Pt-sMoS_2_-RIE based on TEM EDS. (a)** EDS spectrum. **(b)** weight and atomic ratios of Mo, S, and Pt.


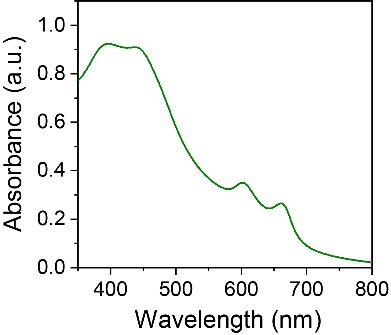


**Figure S9. Absorbance spectrum of sMoS_2_ dispersion measured by an ultraviolet-visible spectrophotometer.**
